# Sanggenon C Stimulates Osteoblastic Proliferation and Differentiation, Inhibits Osteoclastic Resorption, and Ameliorates Prednisone-Induced Osteoporosis in Zebrafish Model

**DOI:** 10.3390/molecules23092343

**Published:** 2018-09-13

**Authors:** Huijuan Wang, Tingting Feng, Donggui Guo, Min Zhang, Lin Chen, Ying Zhou

**Affiliations:** 1 Guizhou Engineering Research Center for the Exploitation and Utilization Technology of Medicine and Food Dual-Use Resources, Guizhou University, Guiyang 550025, China; xuchwhj@163.com (H.W.); dream198896@126.com (M.Z.); edithcl@sina.com (L.C.); 2 College of Pharmacy, Guizhou University of Chinese Medicine, Guiyang 550025, China; ftt0809@163.com; 3 School of Food and Pharmaceutical Manufacture Engineering, Guizhou Institute of Technology, Guiyang 550003, China; cbgdg@163.com

**Keywords:** Sanggenon C, osteoblast, osteoclast, osteoporosis, zebrafish

## Abstract

Sanggenon C (SC), which is a natural flavonoid found in the stem bark of Cortex Mori, has been discovered to have the antioxidant, anti-inflammatory, and antitumor properties. However, its effect in osteoporosis has not yet been reported. In this research, the effect of SC on the proliferation of MC3T3-E1 cells was evaluated by using the MTT assay. Alkaline phosphatase (ALP) activity and the mRNA expression of *Runx2*, *Collagen I*, *OPG*, and *RANKL* were examined. TRAP-positive cell counting and bone resorption pits were adopted to observe the effect of SC on the formation and function of osteoclasts. Next, the mRNA level of *TRAP*, *CTSK*, *NFATc1*, and *TRAF6* of osteoclasts were measured by real-time qPCR. In addition, the anti-osteoporosis activity of SC in vivo was evaluated in the zebrafish model. Our study indicated that SC exhibited a significant stimulatory effect on MC3T3-E1 cell proliferation at 1 to 10 μM and caused an increase in ALP activity at 0.3 to 10 μM. It could upregulate the expression of *Runx2*, *Collagen I*, and increases the *OPG/RANKL ratio*. Furthermore, SC was found to inhibit the formation and function of osteoclasts, which is demonstrated by a lower number of TRAP-positive multinuclear cells and a fewer area of bone resorption pits compared to the control group. *TRAP*, *CTSK*, and *NFATc1* were downregulated in 0.3 to 10 μM SC treated groups. In addition, 3 to 10 μM SC also inhibited the expression of *TRAF6* mRNA. When prednisone-induced zebrafish was treated with 0.3, 1, 3, and 10 μM SC, higher mineralization of vertebrate column was discovered in a dose-dependent pattern, which suggests that SC could reverse the bone loss of zebrafish caused by prednisone. In summary, these findings indicated that SC has the potential to prevent or treat osteoporosis.

## 1. Introduction

Osteoporosis is a systemic bone disease associated with abnormal bone metabolism and reduced bone mineral density. High fracture risk is a common consequence of osteoporosis especially in the aging population [1]. Imbalance in the coupling process between bone dissolution and bone repair is the critical mechanism for osteoporosis. Osteoblasts and osteoclasts are involved as the basic regulatory units and play an important role in the bone remodeling system. Increased osteoclast-induced bone resorption and inadequate osteoblast-mediated bone formation can lead to bone loss and lower bone strength [2,3]. Therefore, the agents targeting osteoblast—osteoclast coupling that inhibit osteoclastic resorption and promote osteoblastic formation may become promising candidates to prevent osteoporosis.

In recent years, natural products have drawn extensive attention because of their specific pharmacological activities and low side-effects. Natural plants are important for new drug discovery [4,5]. Many chemical ingredients isolated from Chinese medicinal plants have been reported to exhibit osteoprotective effect in cell and/or animal models [1,6]. Among these active ingredients, flavonoids are considered to be attractive compounds because of their phytoestrogen-like effects as well as anti-inflammatory and antioxidant properties, e.g., licochalcone A, luteolin, naringin, myricetin, galangin, brazilin, icariin, icaritin, genistein, fisetin, baicalein, etc. [6].

**Figure 1:**
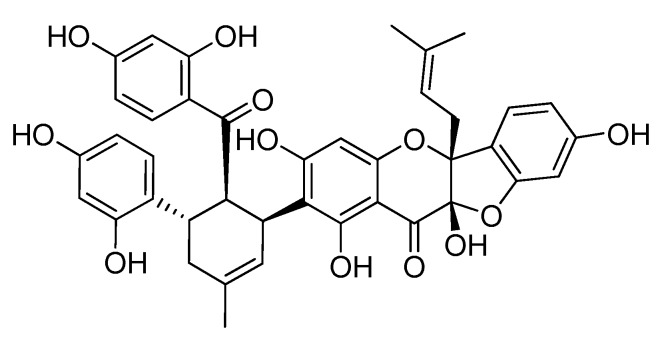
Chemical structure of sanggenon C.

Cortex Mori (Sangbaipi), which is the root bark of *Morus alba* L., is an effective traditional Chinese medicine documented in the Chinese Pharmacopoeia. Our preliminary study found that water extract and ethanol extract of Cortex Mori could stimulate MC3T3-E1 cells and inhibit osteoclast in vitro. Sanggenon C (C_40_H_36_O_12_, Figure 1), which is a main active flavanone originating from Cortex Mori, has been found to possess antioxidant, anti-inflammatory, and antitumor properties [7,8,9,10,11,12]. There was evidence that the increase reactive oxygen species (ROS) level and the inflammatory response were shown to be directly related to the osteoporosis [13,14,15]. Furthermore, it is found that the anti-osteoporosis activity of flavonoids may be attributed to its antioxidant and anti-inflammatory properties. Taken together, we speculated that Sanggenon C may have the potential osteoprotective effect for osteoporosis.

Up to now, there is no available information about Sanggenon C in bone diseases. Therefore, this study was designed to evaluate whether Sanggenon C had the anti-osteoporosis effect and explore its potential mechanism.

## 2. Results

### 2.1. Effect of SC on MC3T3-E1 Cell Proliferation

**Figure 2:**
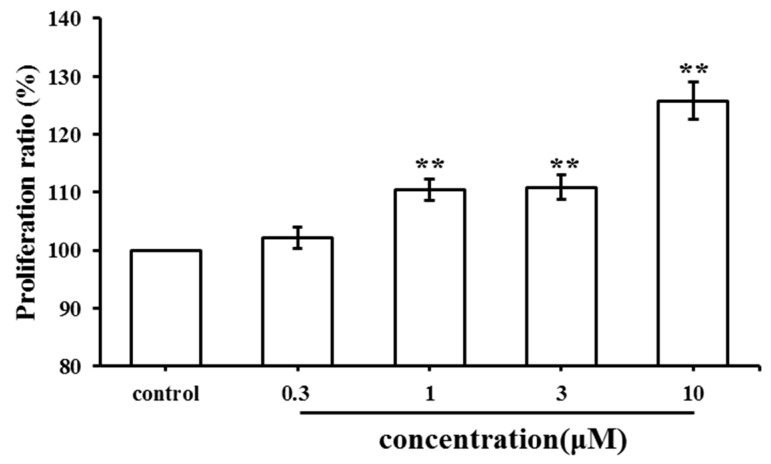
Effect of SC treatment on the MC3T3-E1 cell proliferation (** *p* < 0.01 compared with the control group.

An MTT assay was used to evaluate the effect of SC on cell proliferation. The results were recorded as the proliferation ratio. As shown in Figure 2, SC promoted the proliferation of MC3T3-E1 cells in a dose-dependent manner. Higher doses of SC (1 μM, 3 μM and 10 μM) exhibited a significant stimulatory effect on MC3T3-E1 cell proliferation. The maximum stimulatory effect was achieved when SC was at 10 μM. However, no significant stimulatory effect was observed after treatment with 0.3 μM.

### 2.2. Effect of SC on MC3T3-E1 Cell Differentiation

**Figure 3:**
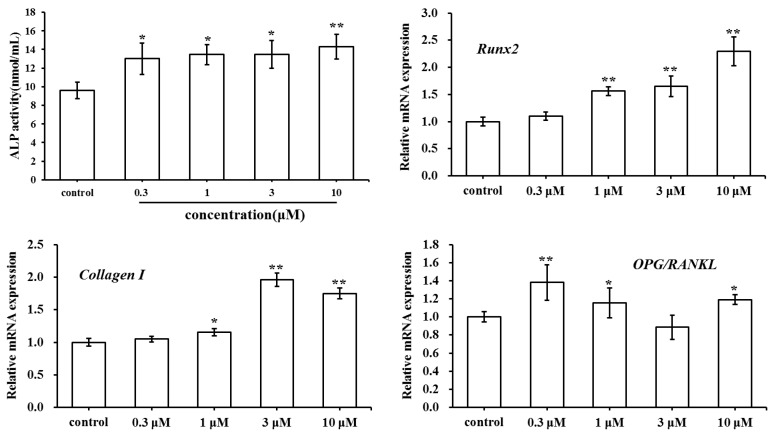
Effect of SC treatment on ALP activity and relative mRNA expression of osteogenic genes (* *p* < 0.05, ** *p* < 0.01 compared with the control group).

MC3T3-E1 cells were exposed to SC for seven days at various concentrations (0.3, 1, 3 and 10 μM). Alkaline phosphatase (ALP) is a phenotypic marker of osteoblastic differentiation. As shown in Figure 3, SC treatment produced a higher ALP activity than the control. SC at 10 μM increased ALP activity significantly (*p* < 0.01 vs. control). By contrast, Runx2 and Collagen I as osteogenic genes were upregulated in the SC-treated group. At the same time, the ratio of OPG/RANKL increased when compared to the control group. These results suggested that SC could promote osteoblast differentiation and also suppress osteoclast growth simultaneously.

### 2.3. Effect of SC on Osteoclast Formation

**Figure 4:**
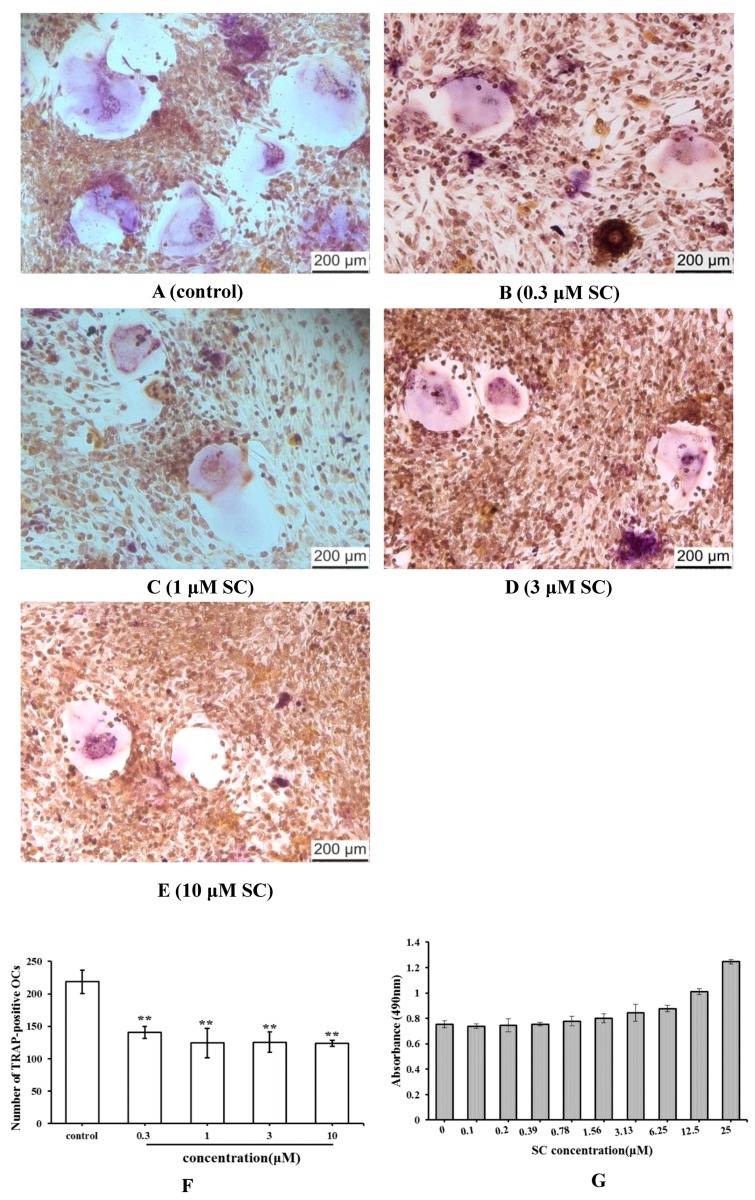
Effect of SC treatment on osteoclast formation (** *p* < 0.01 compared with the control group). (**A**–**E**) TRAP staining images (×100). (**F**) The number of TRAP-positive multinucleated OCs. (**G**) Cell viability assay.

Tartrate-resistant acid phosphatase (TRAP) is an osteoclast-specific symbolic enzyme. To analyze the effect of SC on osteoclast formation, the bone marrow cells induced by 1,25(OH)_2_ VitD_3_ were stained and TRAP-positive cells were counted (Figure 4A–F). By contrast, the osteoclast number of SC group decreased significantly compared to the control group (*p* < 0.01). These findings suggested that SC had the ability to inhibit osteoclast differentiation.

The cell viability was further examined by the MTT method to evaluate whether the osteoclast inhibition of SC was attributed to its cytotoxicity. Consequently, SC was not found to have the cytotoxic effect on bone marrow cells (Figure 4G). These results showed that SC could suppress the formation of osteoclasts from bone marrow cells without any cytotoxic effect.

### 2.4. Effect of SC on Bone Resorption Function of Osteoclasts

**Figure 5:**
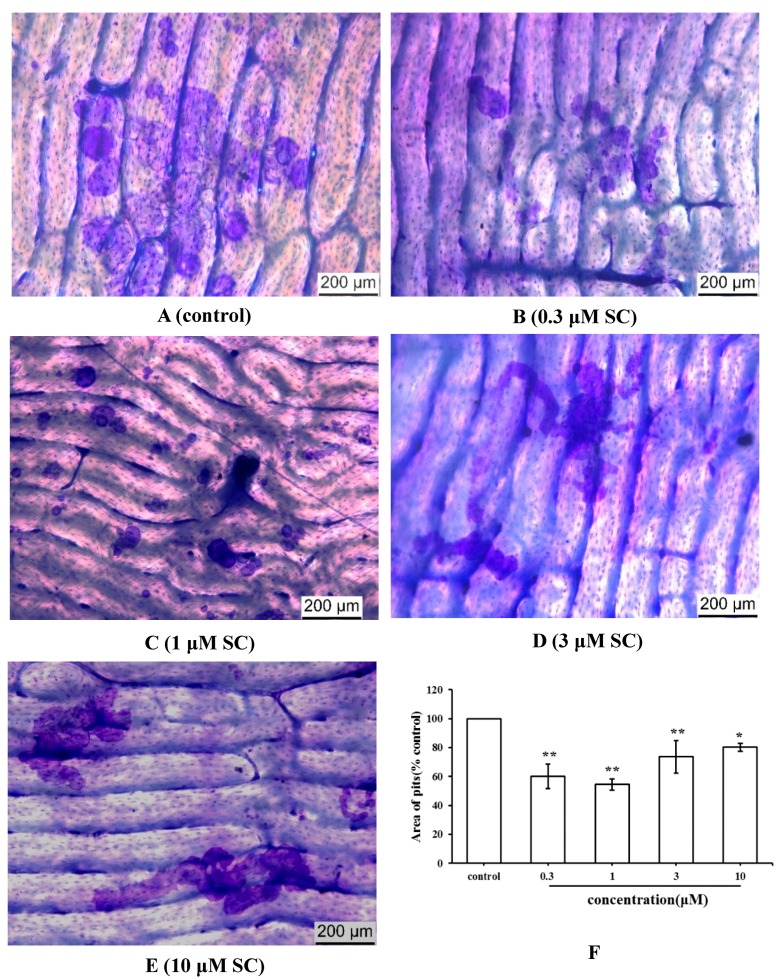
The bone resorption pits formed by rabbit osteoclasts after eight days of incubation (* *p* < 0.05, ** *p* < 0.01 compared with the control group). (**A**–**E**) Pits on the bone slices stained with toluidine blue (×100). (**F**) The ratio of pits area the SC group and the control group.

The bone resorption function of osteoclasts was assayed by measuring the area of pits formed on the bone slices after eight days of incubation. The slices were stained with toluidine blue to identify the resorption pits (Figure 5A–E). The area of pits in the SC group had a lower level than the control group (Figure 5F). These results indicated that SC inhibited the bone resorption activity of osteoclasts.

### 2.5. Effect of SC on the Expression of Osteoclast-Specific Genes

**Figure 6:**
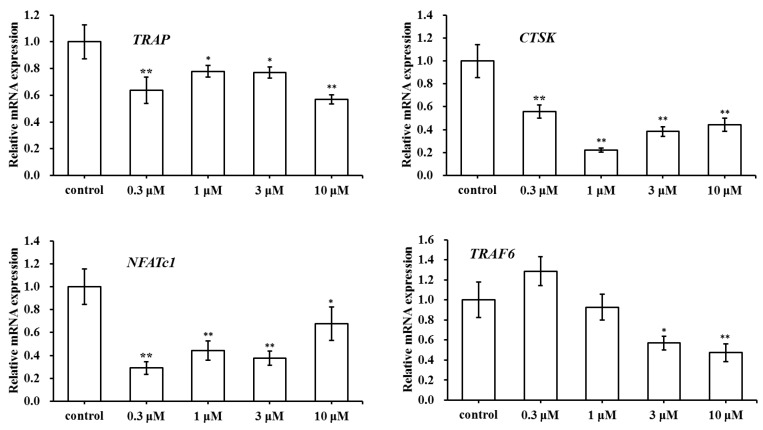
Effects of SC treatment on the relative mRNA expression levels of *TRAP*, *Cathepsin K (CTSK)*, *NFATc1*, and *TRAF6*. * *p* < 0.05, ** *p* < 0.01 compared with the control group.

As an assessment of SC effect on osteoclast formation and activity, we chose a few genes related to bone development including *TRAP*, *CTSK*, *NFATc1*, and *TRAF6*. As illustrated in Figure 6, the mRNA expression of OC genes was determined by real-time qPCR. In contrast with the control group, *TRAP*, *CTSK*, and *NFATc1* were downregulated in SC treated groups at various concentrations (0.3, 1, 3 and 10 μM). SC at concentrations of 3 μM and 10 μM inhibited the expression of *TRAF6* mRNA, but *TRAF6* expression show no significant change in SC treated groups at 0.3 μM and 1 μM. As the results showed, the expression level of *TRAF6* was only inhibited by higher SC concentration. Taken together, the findings suggested that osteoclast-related genes mentioned above were generally inhibited when osteoclasts were treated with SC.

### 2.6. Prevention Effect of SC on Zebrafish Osteoporosis Induced by Prednisone

**Figure 7:**
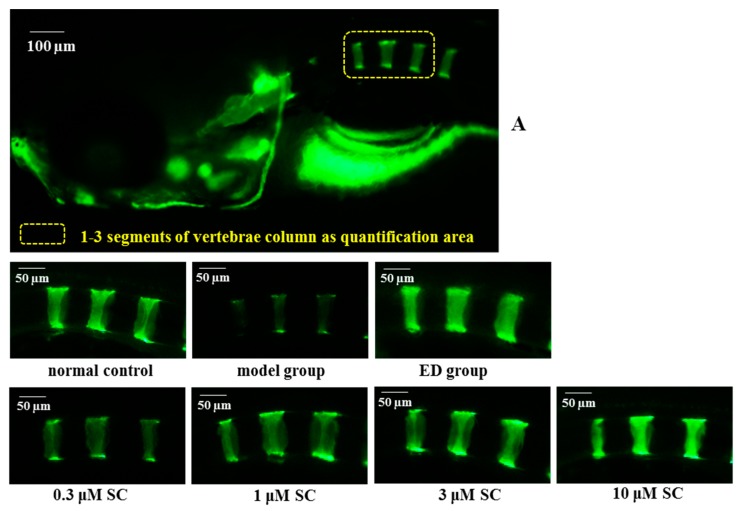
Evaluation of mineralization of the vertebrate column in SC-treated zebrafish. (**A**) The fluorescent images of the calcein-stained vertebrate column in the spinal region of zebrafish (the fluorescence intensity at the area of 1 to 3 segments of vertebrae was detected as quantification of mineralization degree). (**B**) Effect of SC on integrated optical density (IOD) of zebrafish larval (* *p* < 0.05, ** *p* < 0.01 vs prednisone-induced model group, n = 10).

Zebrafish was considered an ideal model organism in biological research and drug screening. In this research, zebrafish embryos at the age of 3 dpf (day-post-fertilization) were incubated with prednisone for five days in microplates to establish a rapid osteopenia model. Etidronate disodium (an anti-osteoporosis drug, ED) was selected as a positive control. The integral optical density (IOD) of 1 to 3 segments of calcein-stained vertebrae was determined to evaluate the amount of bone mineralization of zebrafish larvae. We found that the vertebrate mineralization of prednisone-induced larvae decreased in contrast with the normal control group, which suggested that the osteoporosis model using zebrafish induced by prednisone was successfully developed (Figure 7). When prednisone-induced zebrafish was treated with 0.3, 1, 3 and 10 μM SC, higher mineralization of the vertebrate column was discovered in a dose-dependent pattern. Results also showed that ED could increase the mineralized matrix of the vertebrate column significantly. In conclusion, the study indicated that SC and ED could reverse the bone loss of zebrafish induced by prednisone.

## 3. Discussion

Bone is constantly remodeled by osteoblasts and osteoclasts. Osteoblasts are in charge of bone formation in the maintenance of bone homeostasis. MC3T3-E1 cells cultured in the α-MEM induction medium could exhibit the highly osteogenic differentiation ability. Our study indicated that 1 to 10 μM SC exhibited a significant stimulatory effect on MC3T3-E1 cell proliferation. ALP is a phenotypic marker of osteoblastic differentiation. We observed that SC treatment could produce a higher ALP activity than the control. To elucidate the mechanism, the mRNA expression of osteogenic related genes was examined including *Runx2*, *Collagen I*, *OPG*, and *RANKL*. *Runx2* is essential for osteoblast differentiation as a key transcription factor. It can activate the expression of downstream proteins and induce the differentiation of mesenchymal stem cells into osteoblasts. *Collagen I* is the main composition of bone matrix. It can promote the maturation and mineralization of osteoblasts. *Runx2* and *Collagen I* are referred to as the early markers of osteoblast differentiation. In this study, the expression of *Runx2* and *Collagen I* were found to be upregulated in the SC-treated group, which suggested SC could promote the differentiation of osteoblasts.

*OPG* and *RANKL* are secreted by the mature osteoblasts. They can affect and modulate the differentiation of osteoclasts. *RANKL* is the ligand of *RANK*, which is located on the osteoclast surface. When *RANKL* binds to *RANK*, the complex can stimulate and activate osteoclast differentiation. However, the binding will be suppressed competitively by *OPG*, which is a decoy receptor of RANKL and inhibits the bone resorption. The *OPG/RANKL ratio* is an important indicator for RANKL-activated signaling pathways. The results showed that the ratio of *OPG/RANKL* increased when compared to the control group, which suggests that SC could also suppress osteoclast growth simultaneously.

Osteoclasts, which are derived from bone marrow cells, involved in bone resorption during the process of bone remodeling. In this paper, SC was found to inhibit the formation and function of osteoclasts, which was demonstrated by a lower number of TRAP-positive multinuclear cells and a fewer area of bone resorption pits when compared to the control group. To further illustrate the effect of SC on osteoclast formation and activity, a few genes related to bone development were selected including *TRAF6*, *NFATc1*, *CTSK*, and *TRAP*. *TRAF6* was a major signal transducer for RANKL-activated osteoclastogenesis. The activation of *TRAF6* could trigger various RANKL-related downstream signaling pathways such as NF-κB, MAPKs, and more [16]. *NFATc1* was a master transcription factor for osteoclast differentiation. *TRAP* and *CTSK* were associated with osteoclast function and took part in the bone resorption by destruction of the collagen matrix of the bone. Results indicated that *TRAP*, *CTSK*, and *NFATc1* were downregulated in 0.3 to 10 μM SC treated groups. In addition, SC at concentrations of 3 μM and 10 μM also inhibited the expression of *TRAF6* mRNA. Our results suggested that osteoclast-related genes mentioned above were generally inhibited and SC could suppress the RANKL-RANK signaling pathway.

Above all, the results indicated that SC could stimulate osteoblast and inhibit osteoclast formation and function in vitro, which indicated that SC may exhibit anti-osteoporosis activity. Next, the anti-osteoporosis effect of SC in vivo was evaluated in our study. The zebrafish model could speed up preclinical screening during the drug development pipeline [17]. Now the zebrafish model is rapidly gaining prominence in many aspects of biological and medical research. It has many advantages such as transparent embryos, lots of offspring, and high similarity to human genes [18,19,20,21,22]. In this study, three dpf zebrafish embryos were administrated with prednisone in microplates and a rapid osteopenia model was established. The results showed that higher mineralization of the vertebrate column in SC groups were discovered in a dose-dependent pattern, which suggests that SC could reverse the bone loss of zebrafish caused by prednisone.

## 4. Materials and Methods

### 4.1. Chemicals and Reagents

Sanggenon C (SC, purity, 98.21%) was obtained from the Chengdu Mansite Biotech Co., Ltd. (Chengdu, China). MEM alpha modification was purchased from the HyClone (Logan, UT, USA). Dimethylsulfoxide (DMSO), dexamethasone, and methylt thiazolyl tetrazolium (MTT) were purchased from Solarbio (Beijing, China). Western and IP lysis buffer was obtained from the Beyotime Institute of Biotechnology (Shanghai, China). Fetal bovine serum (FBS) was purchased from Zhejiang Tianhang Biotech Co., Ltd. (Huzhou, China). Minimum essential medium alpha medium (α-MEM) was purchased from gibco (Grand Island, NY, USA). β-glycerophosphate, ascorbic acid, toluidine blue, calcein, TRAP staining kit, and 1,25(OH)_2_ VitD_3_ were purchased from Sigma Chemical Co. (St. Louis, MO, USA). The LabAssay^TM^ ALP assay kit was obtained from Wako Pure Chemical Industries, Ltd. (Osaka, Japan). Prednisone was obtained from the Shanghai Aladdin Bio-Chem Technology Co., Ltd. (Shanghai, China). Etidronate disodium was purchased from Shanghai Yihe Biotech Co., Ltd. (China). The total RNA kit was obtained from OMEGA. Prime Script RT reagent kit and SYBR Green kit for RT-PCRs were purchased from Foregene Biotech Co., Ltd. (Chengdu, China). The oligonucleotide primers were synthesized by Invitrogen Biotech Co., Ltd. (Shanghai, China). Bone slices were obtained from the Institute of Orthopaedics, Lanzhou General Hospital (Lanzhou, China).

### 4.2. MC3T3-E1 Cell Proliferation Assay

Murine MC3T3-E1 cells were obtained from the Shanghai Cell Bank (Chinese Academy of Sciences, Shanghai, China). The cells were cultured in α-MEM containing 10% FBS and 1% penicillin-streptomycin in a condition of 5% CO_2_ and 37 °C.

MC3T3-E1 cells were seeded in 96-well plates at 5000 cells/well. After 24 h of incubation, cells were exposed for 48 h to various concentrations of SC (0, 0.3, 1, 3 and 10 μM). Subsequently, 10 µL MTT (5.0 mg/mL) was added to each well and incubated at 37 °C for another 4 h. Then, the supernatant in each well was discarded and replaced by 150 µL DMSO to dissolve formazan crystals. After shaking for 10 min at room temperature, the absorbance value (OD) was measured at 490 nm on a microplate spectrophotometer (Bio-Rad Model 680, Hercules, CA, USA).

### 4.3. MC3T3-E1 Cell Differentiation

MC3T3-E1 cells were seeded in 12-well plates at a density of 3 × 10^5^ cells/well in α-MEM. After the cells had reached confluence, the culture medium was changed to the osteogenic induction medium (α-MEM supplemented 50 μg/mL ascorbic acid, 10 mM β-glycerophosphate, and 10^−8^ M dexamethasone) containing various concentrations of SC (0, 0.3, 1, 3 and 10 μM). The cells were further cultured for seven days. Then the medium was discarded and each well was gently washed with physiological saline. The cell was lysed and the lysate was centrifuged at 12,000 rpm for 5 min. The supernatant was collected and ALP activity was determined by using the LabAssay^TM^ ALP assay kit.

To elucidate the ability of SC to provide osteoprotective effect, the mRNA expression of osteogenic related genes was examined by real-time qPCR including *Runx2*, *Collagen I*, *OPG*, and *RANKL*.

### 4.4. Culture of Rabbit Osteoclasts

Rabbit osteoclast precursors were prepared as previously described [23]. Bone marrow cells were obtained from the femora and tibiae of two to three day-old rabbits. The resulting cell suspension in α-MEM was filtered through a 200 mesh stainless steel cell strainer. The cells were then seeded in 48-well plates at a density of 2 × 10^6^ cells/mL with or without a sterile bovine bone slice (0.5 cm × 0.5 cm) in each well. After 24 h of incubation, the medium was removed and the cells were gently washed by PBS. To induce osteoclast differentiation, the cells were subsequently cultured in α-MEM medium and supplemented with 10^−8^ M 1,25(OH)_2_ VitD_3_ in the absence or presence of 0.3, 1, 3 and 10 μM SC. Cultures were fed every three days with fresh medium and reagents.

### 4.5. Tartrate-Resistant Acid Phosphatase Staining

After an eight-day osteoclastic induction, the cells were stained using the Leukocyte Acid Phosphatase kit, according to the instruction booklet. The osteoclasts formed were observed under phase-contrast microscopy and TRAP-positive multinucleated cells with ≥3 nuclei considered to be osteoclasts. The total number of osteoclasts per well was counted.

### 4.6. Bone Resorption Assays

After an osteoclastic incubation for eight days, the attached cells were removed by sonication from the bone slices. To identify the resorption pits formed on the slices, the bone slices were stained with toluidine blue (0.1%, *w*/*v*). Images of each slice were obtained under a light microscope and the total areas of resorption pits were determined by Image-Pro Plus 6.0 software (Media Cybernetics, Inc. Rockville, MD, USA).

### 4.7. Relative Expression of Osteoclast-Specific Gene

To investigate the expression of osteoclastic genes, the bone marrow cells were seeded in 12-well plates at 2 × 10^6^ cells/mL and cultured in α-MEM with 1, 25(OH)_2_ VitD_3_ for eight days. The total RNA was extracted and the mRNA expression of *TRAP*, *CTSK*, *NFATc1*, and *TRAF6* were examined by real-time qPCR.

### 4.8. Quantitative Real-Time PCR

The total RNA was extracted from the cells using RNA Kit. It was then converted to cDNA by using RT Easy^TM^ II Reverse Transcriptase. Following cDNA synthesis, real-time qPCR was conducted with Easy^TM^ SYBR Green I Kit on a CFX Connect Real-Time PCR System (Bio-Rad). All the primers used in this paper are listed in Table 1. The amplification conditions were as follows: initial denaturation at 95 °C for 3 min, which was followed by 40 cycles of 10 s at 95 °C, 10 s at 55 °C, and 20 s at 72 °C. The results of PCR reactions were analyzed by the 2^−ΔΔCt^ method.

### 4.9. Animals and Treatments

Wild type zebrafish (AB strain) farming were conducted according to the standard procedure [24]. Adult zebrafish mate and spawn naturally under the condition of 10/14 h dark/light cycle. The zebrafish embryos and larvae were cultured in E3 medium solution (5.0 mM NaCl, 0.17 mM KCl, 0.33 mM CaCl_2_, and 0.33 mM MgSO_4_) under isothermal conditions at 28 °C. The use of zebrafish in this study met the requirement of the institutional Ethical Guidelines for Animal Experiments. The ethic approval number is GZU-201709.

Synchronized embryos at the age of 3 dpf were placed into a six-well plate. Zebrafish larvae were then exposed to 75 μg/mL prednisone and different concentrations of SC (0, 0.3, 1, 3 and 10 μM). In the positive control group, zebrafish were treated with 300 μg/mL etidronate disodium, which is a bone resorption inhibitor. At the same time, zebrafish only treated with medium solution were served as a normal control group. All these groups were incubated at 28 °C until 8 dpf. The medium solution was changed every other day.

### 4.10. Bone Matrix Calcein Labeling

Calcein, which is a fluorescent dye, could bind with calcium in the bone matrix. Calcein labeling could reflect the degree of bone mineralization. Zebrafish larvae aged at 8 dpf was dyed with 0.2% calcein solution for 2 min. Then it was washed with E3 water for 15 min on the shaking table at 50 rpm (repeated three times). The fluorescent images of the calcein-stained vertebrate column were captured under AZ100 fluorescence microscopy (Nikon). The integral optical density of 1 to 3 segments of vertebrae was measured by Image-Pro Plus image analysis software.

### 4.11. Statistical Analysis

All data were recorded as mean ± SD. Statistical analysis was carried out using the SPSS 19.0 software. One-way ANOVA was performed to analyze the group difference. Tukey’s test was conducted for multiple comparisons. *p* < 0.05 means a significant difference and *p* < 0.01 means a highly significant difference.

## 5. Conclusions

In summary, SC exhibited cell-based antiosteoporosis activity through dual action on osteoblasts and osteoclasts in vitro. Further research showed that SC could reverse the bone loss of zebrafish induced by prednisone in vivo. Therefore, SC, which is a natural flavonoid compound, could be developed into a promising anti-osteoporosis agent.

## Figures and Tables

**Table 1:** Primers for real-time qPCR.

Gene	Forward(5′–3′)	Reverse(5′–3′)	bp
**osteoblas**			
*Runx2*	AACTTCCTGTGCTCCGTGCT	CCTGGCTACTTGGTTTTTCA	186
*Collagen I*	CTCCGGCTCCTGCTCCTCTT	CATTGCATTGCACGTCATCG	184
*OPG*	TCTGTGAAAGCAGCGTG	GTTTTGGGAAAGTGGGA	253
*RANKL*	ATGAAAGGAGGGAGCA	AGGGAAGGGTTGGACA	128
β-*actin*	AGGCCAACCGTGAAAAGATG	GGCGTGAGGGAGAGCATAG	185
**Osteoclasts**			
*TRAP*	CGCCAAGCAAATCGGCAAAG	CGGTCACTGAACACGTCCTCGA	141
*Cathepsin K*	GCCTCAAAGTACCCCCG	GTAGCCCCCTCCACAGC	269
*TRAF6*	TGGCTGCCATGAAAAGATGC	TCACGTGGAGGTATGGGAGT	132
*NFATc1*	AGCGTGAACCTGAGGAGTTG	ACCTCCAACATGCGAGCTAC	108
*GAPDH*	AGAGCACCAGAGGAGGACG	TGGGATGGAAACTGTGAAGAG	105
